# Multiparametric MRI based assessment of kidney injury in a mouse model of ischemia reperfusion injury

**DOI:** 10.1038/s41598-024-70401-x

**Published:** 2024-08-27

**Authors:** Soham Mukherjee, Sourav Bhaduri, Rachel Harwood, Patricia Murray, Bettina Wilm, Rachel Bearon, Harish Poptani

**Affiliations:** 1 Centre for Pre-Clinical Imaging, Molecular and Integrative Biology, Institute of Systems, University of Liverpool, Crown Street, Liverpool, L69 3BX UK; 2 Institute for Advancing Intelligence (IAI), TCG CREST, Kolkata, India; 3 Department of Women’s and Children’s Health, Institute of Life Course and Medical Sciences, University of Liverpool, Liverpool, UK; 4 Department of Mathematical Science, University of Liverpool, Liverpool, UK; 5 Department of Mathematics, Kings College, London, UK

**Keywords:** Multiparametric MRI, Ischemia reperfusion injury, Glomerular filtration rate, Dynamic contrast enhanced imaging, Diffusion weighted imaging, Arterial spin labeling, Magnetic resonance imaging, Preclinical research, Acute kidney injury

## Abstract

Kidney diseases pose a global healthcare burden, with millions requiring renal replacement therapy. Ischemia/reperfusion injury (IRI) is a common pathology of acute kidney injury, causing hypoxia and subsequent inflammation-induced kidney damage. Accurate detection of acute kidney injury due to IRI is crucial for timely intervention. We used longitudinal, multi-parametric magnetic resonance imaging (MRI) employing arterial spin labelling (ASL), diffusion weighted imaging (DWI), and dynamic contrast enhanced (DCE)-MRI to assess IRI induced changes in both the injured and healthy contralateral kidney, in a unilateral IRI mouse model (n = 9). Multi-parametric MRI demonstrated significant differences in kidney volume (p = 0.001), blood flow (p = 0.002), filtration coefficient (p = 0.038), glomerular filtration rate (p = 0.005) and apparent diffusion coefficient (p = 0.048) between the injured kidney and contralateral kidney on day 1 post-IRI surgery. Identification of the injured kidney using principal component analysis including most of the imaging parameters demonstrated an area under the curve (AUC) of 0.97. These results point to the utility of multi-parametric MRI in early detection of IRI-induced kidney damage suggesting that the combination of various MRI parameters may be suitable for monitoring the extent of injury in this model.

## Introduction

The prevalence of kidney disease has been a burden on the healthcare system worldwide. The global estimate of the number of patients suffering from chronic kidney disease requiring renal replacement therapy is between 4.9 and 7.08 million^1^. One of the most common causes of acute kidney injury (AKI) is hypoperfusion due to trauma, surgery or sepsis, causing the kidney to become hypoxic; subsequent reperfusion and inflammation causes oxidative stress and further damage to the tissue/organ in a mechanism akin to an ischemia-reperfusion injury (IRI)^2,3^. AKI is a life-threatening condition characterized by loss of kidney function and a rapid decline in the glomerular filtration rate (GFR) which can lead to renal failure^4^. Currently, the clinical diagnosis of AKI relies on a 1.5-fold increase in serum creatinine levels within seven days of injury or oliguria lasting more than six hours^5^, with early interventions including nephric volume expansion, administration of anti-inflammatory and antioxidant drugs, and discontinuation of nephrotoxic medications to prevent further renal injury^6–8^. The pathogenesis of AKI and its long-term effects are not fully understood, but sustained medullary hypoxia is thought to significantly contribute to long-term kidney damage by triggering a cycle of capillary damage, inflammation, and maladaptive tubular repair^9^.

The gold standard measurement of GFR comprises injecting compounds like inulin and chromium ethylenediaminetetraacetic acid (Cr-EDTA). However, these methods are invasive, and require serial blood draws^10–12^. The demand for non-invasive and accurate GFR measurement has led to the development of diverse methods related to the clearance of exogenous markers. These techniques rely on the assessment of the elimination kinetics of fluorescent^13,14^ or radioactive^15^ markers. A significant benefit of these methods is the avoidance of recurrent blood or urine collections and laboratory analyses. Transcutaneous GFR measurement devices have demonstrated their efficacy for global GFR estimation and validation in rodent models^16–18^.

There is also a growing need for independent measurement of kidney function (left and right kidney separately) since occasionally only one kidney might be damaged with the other compensating for the loss of renal function in the damaged kidney. Current techniques of measuring individual kidney GFR include nuclear imaging methods using contrast agents such as DMSA^19^, MAG3^20^ and DTPA^21^. Magnetic resonance imaging (MRI), on the other hand, has an intrinsic advantage over these methods as it is non-ionizing and can be used in longitudinal studies for assessing kidney damage and therapeutic response^22^. Functional MRI can also be used to measure individual kidney GFR^23^.

Diffusion weighted imaging (DWI) MRI can be used to detect IRI damage to renal tissue by measuring changes in the microstructure of the tissues^24^. In IRI, there is a disruption of renal microcirculation, which can lead to tissue hypoxia and subsequent cellular swelling, necrosis, and apoptosis impacting the diffusion of water molecules, which can be detected by DWI. In IRI, fibrosis and inflammation can reduce the extracellular space, leading to a decrease in magnitude of diffusion of the water molecules which is measured by Apparent Diffusion Coefficient (ADC) values^25^. DWI can detect the changes in ADC values, which can indicate the extent and severity of IRI damage to renal tissue. Ding et al. reported a positive correlation between ADC and GFR in patients with severe renal injury^26^. Besides measuring tissue microstructure with standard DWI, the Intra-Voxel Incoherent Motion (IVIM) analysis of the DWI data using a biexponential fitting can also provide D or True diffusion, D* or the pseudodiffusion coefficient, and f or the perfusion fraction values. These parameters have been reported as markers of renal injury^27^.

Arterial spin labeling (ASL) involves capturing a labeled image and a control image, both having similar static tissue signals but distinct magnetization in inflowing blood. By magnetically labeling water molecules in arterial blood with a radiofrequency pulse, the subtraction of labeled from control images isolates signals that are linearly linked to tissue perfusion, provides insights into renal blood flow (RBF). ASL MRI has been increasingly used in animal models to study the pathophysiology of renal diseases and to evaluate efficacy of potential therapies^28–31^. If the kidney recovers, cortical perfusion will increase, whereas if the kidney deteriorates, cortical perfusion will decrease^32,33^. ASL MRI has been used to demonstrate that by keeping the renal pedicle occlusion time constant, decrease in perfusion in injured kidneys varies with the mouse strain^29,31^. Niles et al. demonstrated advantages of monitoring the cortical perfusion through ASL MRI^34^. ASL MRI has also been shown to be sensitive to differentiate damage between moderate and severe AKI^31,32^.

Dynamic contrast enhanced (DCE) MRI works by introducing gadolinium-based contrast agent into the bloodstream and rapidly imaging the tissue of interest. The generated image sets can be analyzed using pharmacokinetic (PK) model fitting to estimate the physiological functions of the tissue. Several PK models have been proposed to analyze renal DCE-MRI data^35–38^. However, no consensus has been established on the most relevant/accurate model as the models used for the analysis of renal DCE-MRI data typically tend to overestimate the GFR^23,39–41^. The lack of a standardized PK model leads to variability across studies^42–45^. Empirical models can reduce the variability by utilizing a data driven fitting approach. Empirical models optimize parameters by taking advantage of multiple models according to the data characteristics. The principle of parsimony dictates that, when all factors are equal, a preference is given to simpler models with fewer parameters over more intricate, highly parameterized ones^46^. Nevertheless, complex models frequently provide superior fits to experimental data. Achieving the best-fit model necessitates a delicate equilibrium between goodness of fit and model complexity to prevent overparameterization.

Multiparametric MRI holds promise as a comprehensive tool for assessing both the structural and functional aspects of renal health^47–50^. Its application extends to the early detection of renal abnormalities and pathological changes, facilitating timely intervention and effective management. The multiparametric MRI approach yields quantitative data, ensuring precise and objective measurements of various renal parameters in individual kidneys. This quantitative aspect is pivotal for accurate assessment and the longitudinal tracking of changes in renal conditions. In the early stages of AKI (within 7 days), there is a significant impact on the vasculature and tubular function of the kidney. Ischemia induced during the injury process can be monitored using DCE-MRI to assess filtration and using ASL and IVIM-DWI to evaluate blood and tubular flow. The reduction in blood flow also leads to capillary damage, which can be monitored using ASL and IVIM-DWI. Additionally, ischemia increases reactive oxygen species (ROS) production, leading to cell inflammation and swelling, which subsequently reduces water diffusion. This reduction can be mapped using DWI-MRI. Since a single MRI technique can only monitor one aspect of the injury/repair process, it is essential to assess multiple aspects of pathophysiology to gain a comprehensive understanding of injury recovery or progression and to effectively monitor treatment responses.

Thus, the primary objective of this study was to evaluate the effectiveness of multi-parametric MRI, utilizing DCE-MRI, ASL, and DWI-derived parameters, in detecting IRI-induced kidney damage. To achieve this aim, we employed a parsimonious model for estimating GFR from DCE-MRI and cross-validated the results with a transcutaneous GFR measurement device. The MRI-GFR value was subsequently used to monitor filtration, while ASL and DWI were employed to monitor perfusion and diffusion, respectively. Using a multiple linear regression analysis approach, the three MRI methods were used to track changes in kidney injury/recovery over time in a unilateral IRI rodent model at baseline, day 1, and day 15 post-injury.

## Results

**Figure 1:**
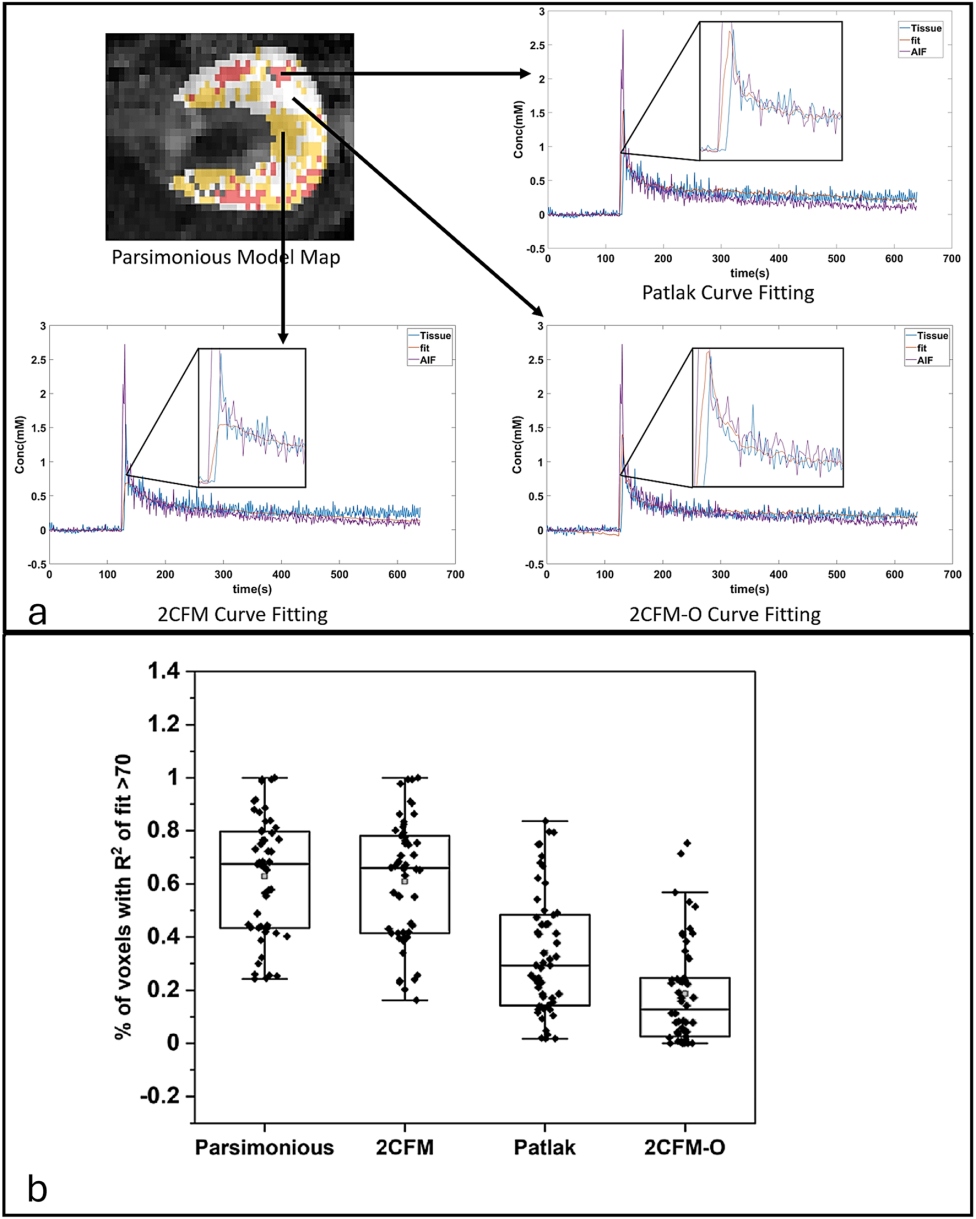
(**a**) Representative parsimonious PK model derived F_T_ map overlaid on a T2 weighted image. Individual concentration-time curves from representative areas using Patlak, 2CFM and 2CFM-O models are also shown. (**b**) Box plots showing the comparisons of percentage of voxels with R^2^ higher than 70 with the 3 PK models (Patlak, 2CFM, 2CFM-O) and the parsimonious PK model.

To demonstrate the efficacy of the parsimonious model over the Patlak, 2CFM and 2CFM-O models, all the acquired DCE-MRI datasets were analysed. Representative fitting of the data using the parsimonious model with voxels from the three individual PK models are shown in (Fig. 1a). The percentage of voxels having R^2^ higher than 70% are shown in (Fig. 1b) which shows that the parsimonious model has the highest mean percentage of voxels with R^2^ higher than 70% followed by 2CFM, Patlak and 2CFM-O.

**Figure 2:**
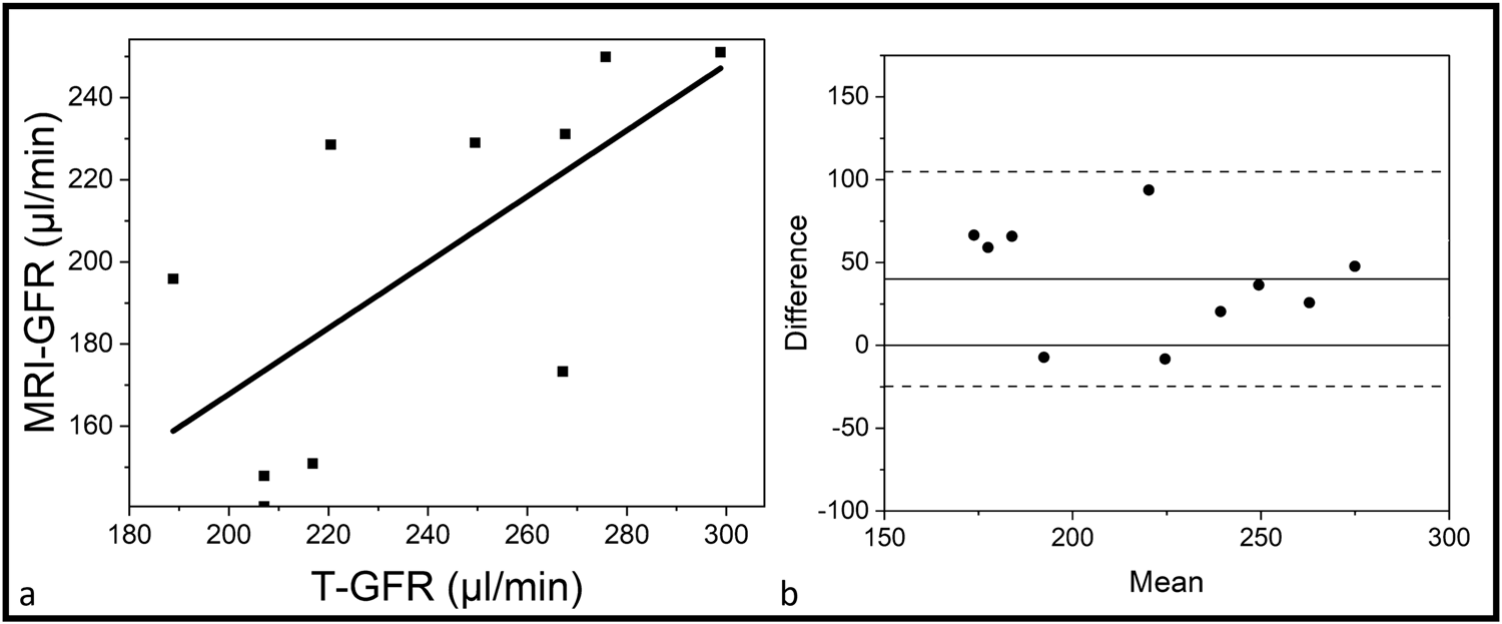
(**a**) Correlation plot between Transcutaneous GFR and MRI GFR (R^2^ = 0.44, p = 0.03) (**b**) Bland–Altman plot between the GFR measurement methods.

After establishing the efficacy of the parsimonious model in terms of fitting ability, our aim was to establish its usability in assessing kidney GFR. A separate cohort of ten healthy mice underwent DCE-MRI followed by transcutaneous GFR measurement to cross-validate the utility of DCE-MRI in assessing kidney GFR. DCE-MRI derived GFR values (parsimonious model derived F_T_ X kidney volume), were cross-correlated with the GFR values calculated using a transcutaneous device. Figure 2a. shows a strong and positive correlation between transcutaneous GFR and MRI based GFR (R^2^ = 0.44, p = 0.03). No proportional biases in the two measurements were noted as demonstrated in the Bland Altman plot shown in (Fig. 2b).

**Figure 3:**
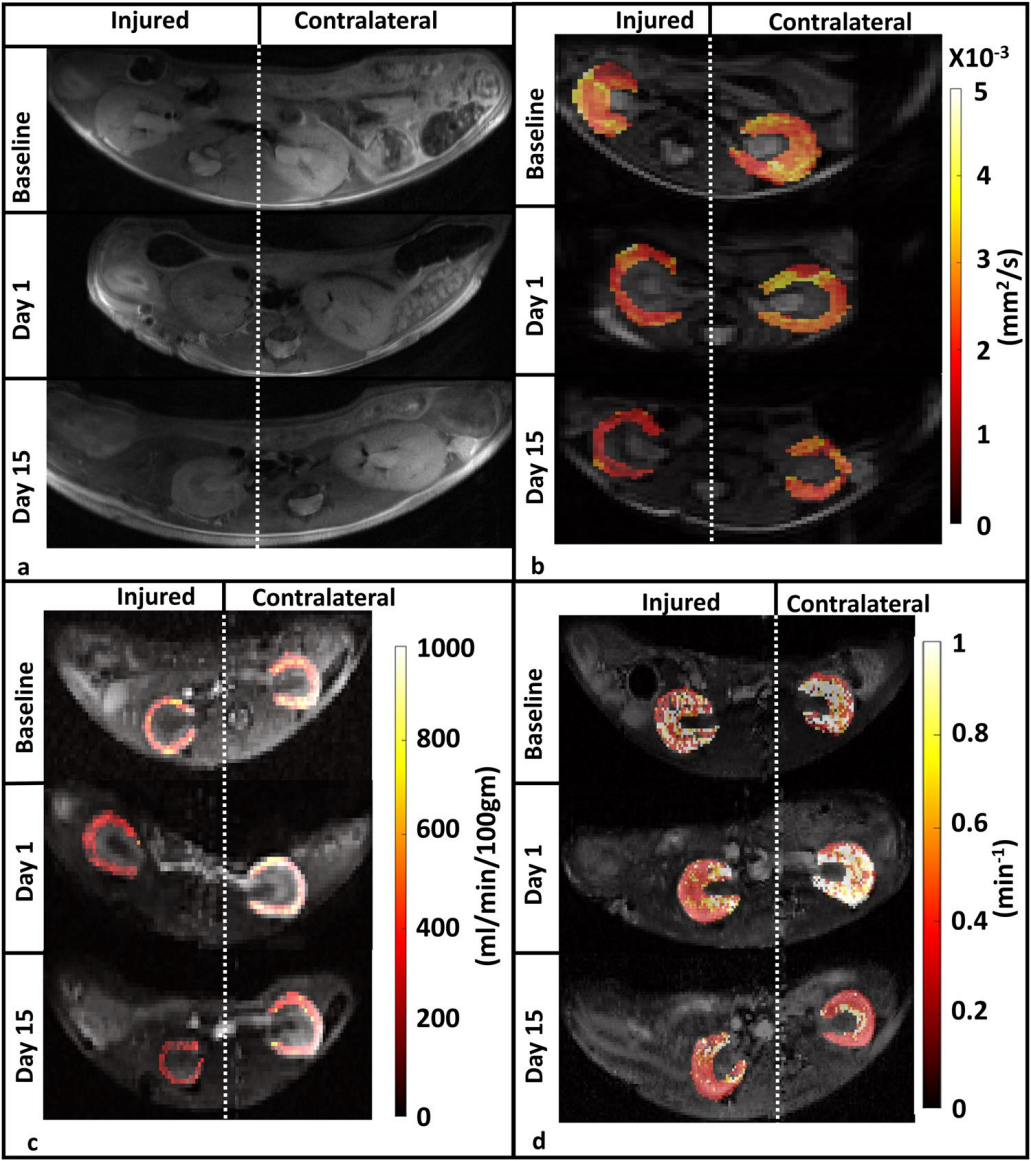
Representative images from mice imaged using MRI at baseline and on day 1 and 15 of the IRI experiment. (**a**) T2 weighted Images of left (contralateral) and right (injured) kidneys of a representative animal. (**b**) ADC maps of left and right kidneys of a representative animal show clear decrease in ADC of the injured kidney at days 1 and 15. (**c**) ASL Maps of left and right kidneys of a representative animal show clear decrease in RBF of the injured kidney at days 1 and 15. (**d**) ${F}_{T}$ maps derived from the parsimonious fitting of the of left and right kidneys of a representative animal show clear reduction in filtration between left and right kidney at day 1. This difference was less obvious at day 15.

After demonstrating a good correlation between the parsimonious model derived GFR and GFR calculated using transcutaneous GFR device, we evaluated the ability of multiparametric MRI to differentiate between healthy and the injured kidney after IRI injury. A different cohort of nine mice underwent multi-parametric MRI on baseline (where both kidneys were assumed to be healthy), day 1 after IRI surgery (right kidney was clamped (injured) and left kidney was used as an internal control/contralateral) and day 15 after IRI surgery (right kidney is injured and left kidney is internal control/contralateral). Representative example cases showing the changes in renal parameters from baseline, day 1 and day 15, in the left (contralateral) and right (injured) kidneys after IRI surgery are shown in (Fig. 3).

**Figure 4:**
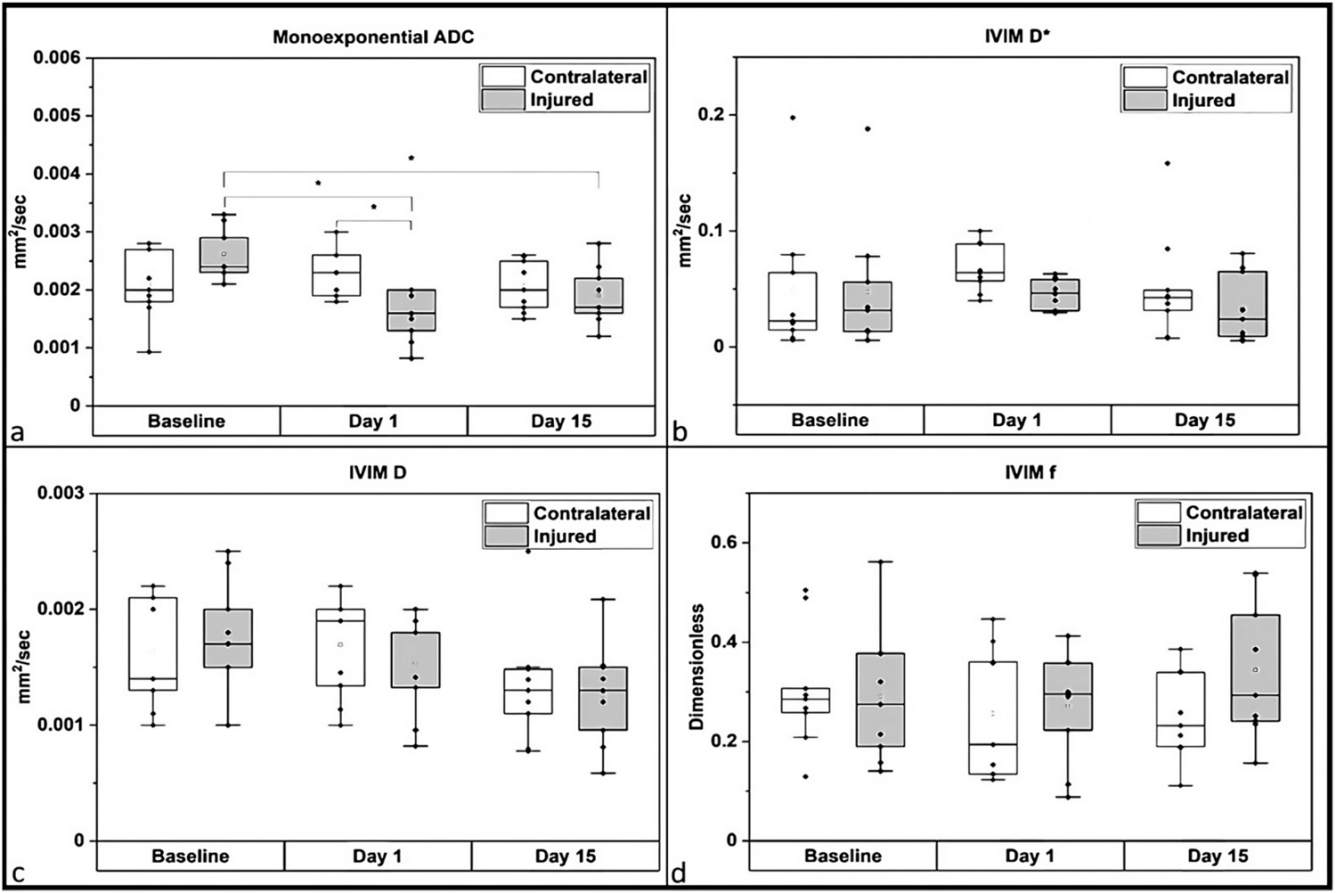
Boxplots showing longitudinal changes in the IRI right/injured (grey) and left/contralateral (white) DWI parameters (**a**) apparent diffusion coefficient (ADC) using the mono-exponential fitting of the DWI data (**b**) pseudo-diffusion coefficient (D*), (**c**) pure diffusion coefficient (D) (**d**) perfusion fraction (f) measured using the IVIM fitting of the DWI data.

Dynamic changes in the kidney tissue microstructure after IRI injury are shown in (Fig. 4). Typically, lower ADC and D values denote reduced extracellular space due to fibrosis and inflammation, while D* and f values reflect the micro perfusion in the tissues.

Significantly lower ADC values were observed in the injured kidneys compared to the contralateral kidney on day 1 post IRI (p = 0.048) (Fig. 4a). Furthermore, a significant reduction in the ADC of the injured kidneys compared to baseline values was also observed on day 1 (p < 0.001) and day 15 (p < 0.05). However, none of the IVIM-DWI derived parameters including D, D* or f demonstrated any significant changes between the injured and contralateral kidneys or even longitudinal changes compared to baseline values (Fig. 4b–d).

**Figure 5:**
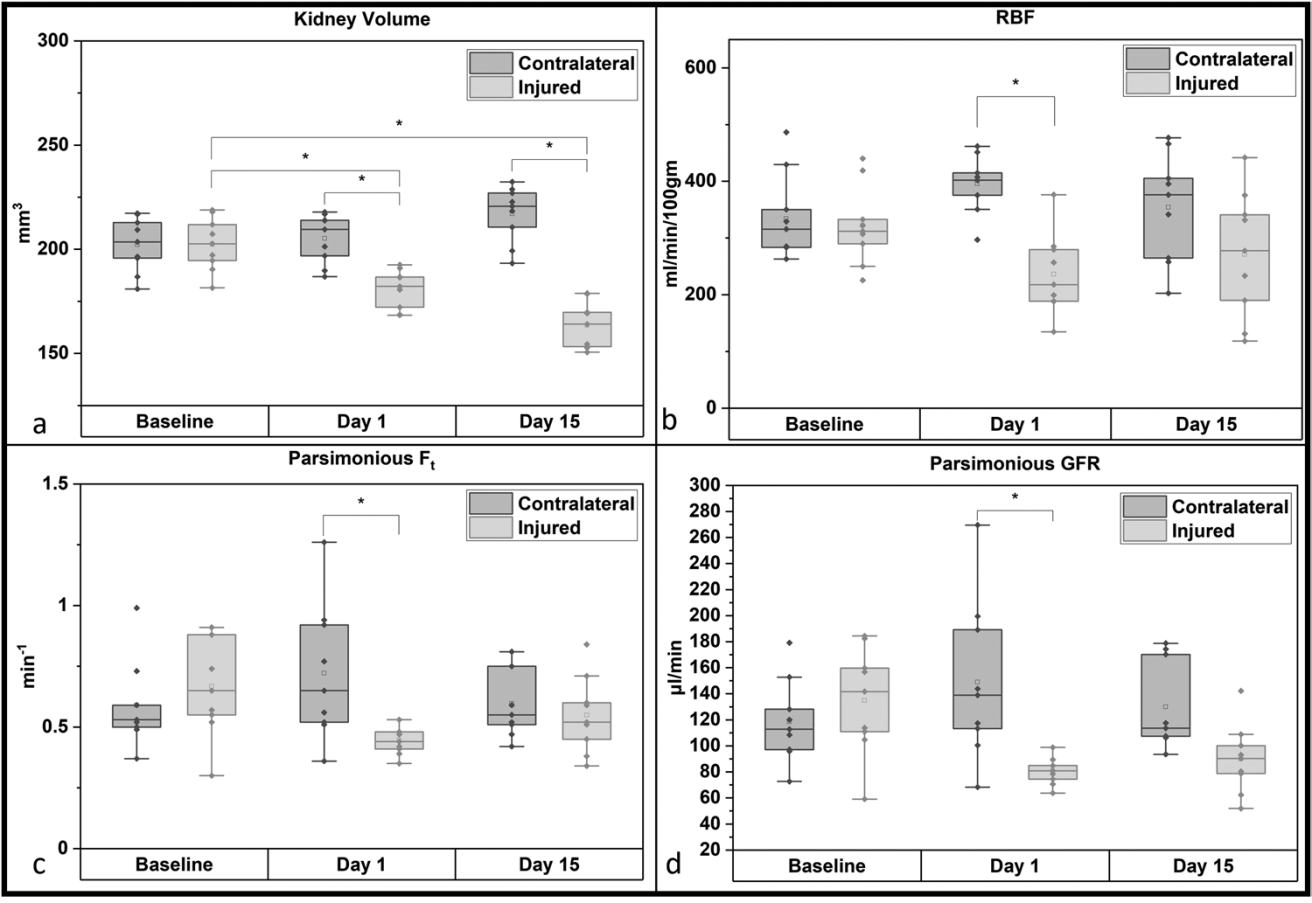
Boxplots comparing the left and the right kidney at baseline (n = 9) with IRI (n = 9) right/injured (grey) and left/contralateral (white) kidney parameters (**a**) Kidney Volume (**b**) RBF, measured via ASL technique (**c**) ${F}_{T}$ measured using parsimonious model (**d**) GFR calculated from ${F}_{T}$ measured using parsimonious model.

Fig. 5a shows changes in kidney volume after IRI surgery. Significantly smaller kidney volume was noted in the injured kidney after IRI surgery than that of the contralateral kidney (p = 0.001), which was also significantly lower than the baseline values measured prior to IRI surgery (baseline scans (p = 0.006)). However, there were no significant changes in the contralateral kidneys volume between baseline and day 1. Fifteen days post-IRI surgery, the injured kidney volume was significantly lower than the contralateral kidney (p <  < 0.001), which were also significantly lower compared to baseline (p <  < 0.001) values. However, the contralateral kidney demonstrated a trend of increasing volume although these changes were not significant over the 15 day period post IRI injury.

Similar to volume changes, significant reductions in the renal blood flow (RBF) values were noted in the injured kidney (Fig. 5b). On day 1 post IRI, the RBF of the injured kidney was significantly lower than the RBF of the contralateral kidney (p = 0.002). However, by day 15 post-IRI surgery, the injured kidney RBF values were similar to the baseline values.

The parsimonious model derived F_T_ and subsequently derived GFR values from the kidneys of these animals are shown in (Fig. 5c,d). A significantly lower filtration coefficient (${F}_{T}$, p = 0.038) and GFR (p = 0.005) value of the IRI induced injured kidney was observed compared to these values in the contralateral kidneys on day 1 post-IRI surgery. However, by day 15 post-IRI surgery, ${F}_{T}$, and DCE-GFR values were similar to the baseline values.

Table 1 shows the group data from all the 9 animals used in the IRI experiment showing changes in the contralateral and injured kidneys with respect to volume, renal blood flow, parsimonious model derived F_t_ and the DCE-MRI derived GFR values.

**Table 1:** Parametric values from the IRI experiment showing dynamic changes in parameters. (a) Baseline values (b) Day 1 after surgery (c) Day 15 after surgery.

Parameter	Mean ± SEM		C.I	
	Right	Left	Right	Left
(a) Baseline				
Volume (mm^3^)	202.46 ± 4.24	202.19 ± 4.36	[192.68, 212.25]	[192.15, 212.24]
RBF(ml/min/100gm)	322.02 ± 23.37	333.92 ± 25.81	[268.12, 375.93]	[274.38, 393.45]
ADC (m^2^/s)	0.0026 ± 0.00015	0.0021 ± 0.0002	[0.0023, 0.0030]	[0.0016, 0.0026]
D (m^2^/s)	0.0017 ± 0.00018	0.0016 ± 0.00016	[0.0013, 0.0021]	[0.0013, 0.0020]
D* (m^2^/s)	0.047 ± 0.019	0.049 ± 0.02	[0.0029, 0.092]	[0.0019, 0.096]
f	0.29 ± 0.045	0.3 ± 0.04	[0.19, 0.39]	[0.21, 0.4]
Pars F_T_ (min^−1^)	0.67 ± 0.068	0.59 ± 0.059	[0.51, 0.82]	[0.45, 0.73]
Pars GFR (μl/min)	134.84 ± 13.7	118.58 ± 10.64	[103.23, 166.44]	[94.04, 143.12]

Parameter	Mean ± SEM		C.I	
	Right/injured	Left/contralateral	Right/injured	Left/contralateral
(b) Day 1				
Volume (mm^3^)	180.97 ± 3.08	205.18 ± 3.96	[173.86, 188.08]	[196.06, 214.31]
RBF(ml/min/100gm)	236.25 ± 23.84	395.92 ± 16.76	[181.27, 291.23]	[357.09, 434.41]
ADC (m^2^/s)	0.0016 ± 0.00015	0.0022 ± 0.00015	[0.0012, 0.0019]	[0.0020, 0.0026]
D (m^2^/s)	0.0015 ± 0.00014	0.0017 ± 0.00015	[0.0012, 0.0019]	[0.0013, 0.0020]
D* (m^2^/s)	0.045 ± 0.0045	0.068 ± 0.0069	[0.035, 0.056]	[0.052, 0.084]
f	0.27 ± 0.037	0.26 ± 0.044	[0.19, 0.35]	[0.15, 0.36]
Pars F_T_ (min^−1^)	0.44 ± 0.02	0.72 ± 0.09	[0.4, 0.49]	[0.51, 0.94]
Pars GFR (μl/min)	80.66 ± 3.47	148.94 ± 20.41	[72.64, 88.67]	[101.88, 195.99]

Parameter	Mean ± SEM		C.I	
	Right/injured	Left/contralateral	Right/injured	Left/contralateral
(c) Day 15				
Volume (mm^3^)	163.51 ± 3.74	217 ± 4.47	[154.90, 172.13]	[206.69, 227.31]
RBF(ml/min/100gm)	271.1 ± 37.18	354.01 ± 31.77	[185.36, 356.84]	[280.74, 427.28]
ADC (m^2^/s)	0.0019 ± 0.00017	0.0021 ± 0.00015	[0.0015, 0.0023]	[0.0017, 0.0024
D (m^2^/s)	0.0013 ± 0.00015	0.0013 ± 0.00017	[0.0009, 0.0016]	[0.0009, 0.0017]
D* (m^2^/s)	0.033 ± 0.01	0.051 ± 0.015	[0.011, 0.057]	[0.016, 0.087]
f	0.34 ± 0.047	0.25 ± 0.03	[0.24, 0.45]	[0.18, 0.32]
Pars F_T_ (min^−1^)	0.55 ± 0.05	0.6 ± 0.05	[0.43, 0.67]	[0.49, 0.70]
Pars GFR (μl/min)	89.77 ± 8.84	129.9 ± 11.36	[69.39, 110.15]	[103.71, 156.09]

**Figure 6:**
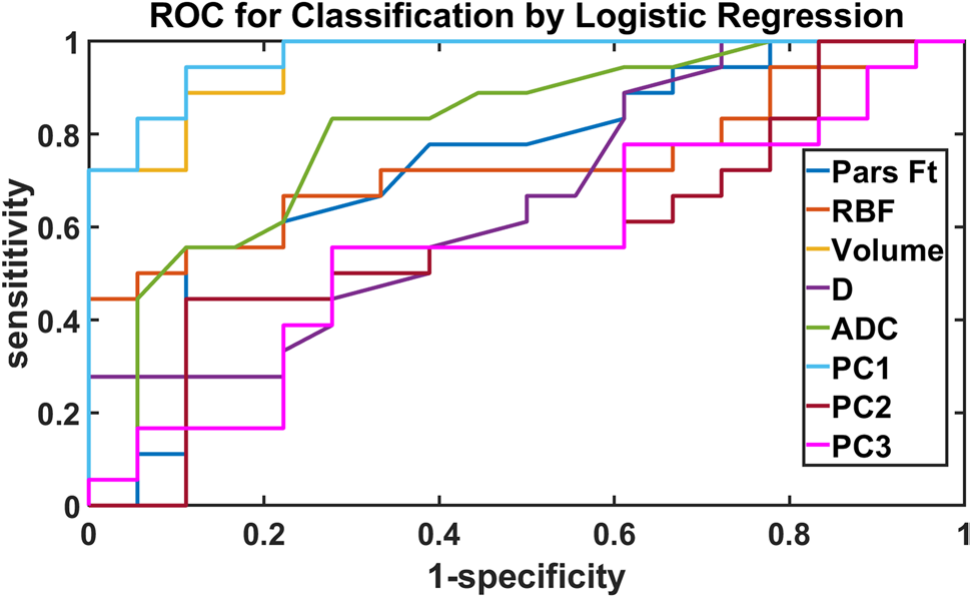
Receiver operating characteristics curves using univariate and multivariate analysis of the MRI data derived parameters for individual parameters (excluding D* and f) and the 3 principal components (AUC of PC1—0.97, AUC of PC2—0.58, AUC of PC3—0.56).

Univariate ROC analysis was performed to test the sensitivity and specificity of the MRI derived parameters for the difference between injured (day 1 or day 15) and healthy kidney (baseline values). Figure 6 and Table 2 show the receiver operating characteristic (ROC) curves and corresponding area under the curve (AUC) values. Significant differences between the measurements obtained for injured and contralateral kidneys were seen in ${F}_{T}$, RBF, kidney volume, and ADC with an AUC > 0.7. However, D* and f from IVIM had an AUC < 0.5 and hence were not included in Fig. 6. Multivariate logistic regression was then conducted through a principal component analysis (PCA) utilizing parameters demonstrating an AUC > 0.5. Table 2 shows the five principal component coefficients, cumulative variance (CV) and individual AUC from the ROC analysis. As, PC1-PC3 had a CV of 80%, it was used for ROC analysis which demonstrated that PC1 was the most discriminative component, with an AUC of 0.97, the coefficients of the parameters along the three principal components are shown in (Table 2).

**Table 2:** Coefficients of parameters along 5 principal components and their respective AUC.

Parameters	PC1	PC2	PC3	PC4	PC5	AUC
Pars ${F}_{T}$	0.32	0.63	−0.47	0.15	0.50	0.73
RBF	0.30	0.45	0.75	0.35	−0.15	0.73
Vol	0.54	−0.02	0.20	−0.81	0.12	0.96
D	0.39	−0.62	0.14	0.39	0.54	0.66
D*	–	–	–	–	–	0.44
f	–	–	–	–	–	0.47
ADC	0.60	−0.13	−0.39	0.21	−0.65	0.81
CV	36.7%	62.85%	79.94%	93.08%	100%	

## Discussion

In this study, we demonstrated that multi-parametric MRI can detect differences between IRI induced injured and healthy kidney. More specifically, F_T_, RBF, kidney volume and ADC can serve as indicators for evaluating kidney damage and potentially as markers for evaluating treatment response.

The selection of the most appropriate pharmacokinetic model holds significant importance in the analysis of DCE-MRI data. Using an optimal PK model requires not only a detailed understanding of the underlying physiological process but also needs to strike a careful balance in fitting the acquired data and ensuring precision in the derived parameter values. Renal DCE-MRI studies in mice are challenging due to respiratory motion induced artifacts and partial volume effects in measuring the kidney volume. Addressing these challenges while striving for higher temporal resolution impacts the measurement of arterial input function (AIF) as well as pharmacokinetic model fitting^51^. Consequently, variations in acquisition parameters and data quality arise, contributing to uncertainty regarding the optimal model for analyzing DCE-MRI^52,53^. The dilemma stems from the acknowledgment that intricate models with multiple parameters may mimic physiology but pose fitting challenges in the presence of low data quality. Using an Akaike criterion based parsimonious model, we demonstrated (Fig. 1b) that the ${F}_{T}$ values can be reproducibly measured from the mouse kidney with most voxels demonstrating a good fitting of the PK model curve. Beeman et al.^54^ have used a similar approach using a Bayesian probability theory for mouse renal DCE-MRI demonstrating the advantages of using an empirical model in conjunction with pharmacokinetic models in the analysis of renal function.

By utilizing the ${F}_{T}$ values derived from the parsimonious model, a significantly positive correlation was observed between MRI based GFR and GFR values measured using a transcutaneous device. Further exploration of the agreement between these two methods using a Bland Altman plot analysis revealed that the limits of agreement range from -24.70 to 104.85 µl/min, indicating variability in the differences between the two methods. Despite this variability, MRI-GFR shows a reasonable level of precision as all the datapoints lie within the limits of agreement. Our mean difference (bias) of [(T-GFR)—(MRI-GFR)] is 40.08 µl/min, indicating that MRI-GFR tends to underestimate GFR compared to T-GFR.

While MRI-GFR underestimates GFR values compared to transcutaneous GFR, it is unclear whether this underestimation holds true when compared to gold standard GFR values derived from repeated blood sampling techniques. Should the systemic underestimation persist, it can be addressed by applying a calibration factor and refining MRI techniques. To determine the appropriate calibration factor, a future comparative study should be conducted using a large cohort of animals, preferably larger animals where repeated blood sampling will not impact blood pressure and GFR, to evaluate MRI-GFR, transcutaneous GFR, and gold standard GFR. Meanwhile, MRI-GFR values can provide GFR values similar to those obtained through the transcutaneous device, with the added advantage of demonstrating spatial differences in individual kidneys as compared to eGFR values, that can only provide a global estimate of the GFR from both kidneys.

A significant reduction in kidney volume after one day of IRI surgery and after 15 day IRI injury is in agreement with earlier findings^25,55^, which confirms that unilateral kidney injury causes kidney volume reduction contributing to the loss of renal function. Induction of IRI through renal artery clamping leads to a reduction in perfusion, underscoring the importance of monitoring perfusion for assessing kidney function. On day 1 post-IRI surgery, perfusion (RBF) in the injured kidney significantly decreased compared to the contralateral kidney which has also been previously reported^30^. However, by day 15 after IRI, no significant differences in RBF was observed between the injured and uninjured kidneys. Using the same strain of mice as used in our study, Hueper et al.^30^ suggested a recovery of renal function after 15 days with a IRI clamping time of 35 min, while a clamping time of 45 min led to a continued deterioration in perfusion in the injured kidney. Since we used a clamping time of 40 min, it is possible the RBF values may have been impacted by the intermediate clamping time and hence the varied recovery of the injured kidney after 15 days of IRI. A similar pattern was evident in the parsimonious model-derived ${F}_{T}$ parameter in our study, whereby a notable decrease in ${F}_{T}$ was observed in the injured kidney compared to the contralateral kidney on day 1 post IRI. However, no such distinctions were apparent on day 15. The GFR of the injured kidney at day 15 was not significantly different from baseline values, which is at variance with an earlier study by Jiang et al.^45^, which used the 2CFM-O model for the PK analysis of the DCE data. This may be due to the difference in the IRI model used in the two studies as we used a 40 min arterial clamp while Jiang and colleagues used a renal arterial stenosis model which causes a significantly more severe injury to the kidney. Of note, no significant differences in F_T_ between the clamped and the control kidneys were observed after 24 h when the 2CFM model was used^56^. Collectively, previously published studies indicate that depending on the PK model used, variabilities in the F_T_ and derived GFR values may exist. However, a parsimonious data driven approach as used in this study may partly address this variability, at least with regards to the PK model being used to analyze the DCE-MRI data.

Significant reductions in the ADC of injured kidney were noted after 1 day of IRI injury as compared to the contralateral kidney cortex using ADC values from the mono-exponential fitting of DWI data. These findings are consistent with findings by Hueper et al.^57^ and Greite et al.^58^, who reported similar decrease in ADC values from the kidney cortex after unilateral IRI. However, 15 days after IRI surgery, we noticed that the ADC value of the injured kidneys increased slightly, although they were still significantly lower than their baseline values. We did not observe any significant differences in the diffusion parameters estimated using biexponential modelling of IVIM-DWI data. However, Cheung et al.^24^ reported significant differences in true diffusion (D) and pseudodiffusion/perfusion fraction (f) in a rat model of unilateral renal IRI where a 60 min clamping duration was used and the changes in diffusion values were assessed 5 h post IRI. The apparent differences between our study and by Cheung et al.^24^ might be due to the severity of the injury as well as the difference in the imaging time points between the two studies.

Using a multi-parametric analysis, we were able to determine that kidney volume, ADC, RBF, ${F}_{T}$ and MRI-GFR can be used to differentiate between the injured and the healthy kidney. However, when univariate analysis was performed, only kidney volume and ADC showed significant reduction in the injured kidney compared to their baseline (healthy) values. To further investigate if any other parameter has the potential to differentiate between healthy and injured data, a binary classifier ROC analysis was performed taking baseline data from left and right kidney as healthy and taking injured kidney data from day 1 and day 15 as injured. MRI-GFR was used in this analysis as it is a product of ${F}_{T}$ and kidney volume. ROC analysis revealed that although IVIM D by itself didn’t show any significant difference between healthy and injured kidney, it can still contribute to the multi-parametric analysis in differentiating between healthy and injured kidney. Further to estimate the combination of parameters which can be used to differentiate between healthy and injured kidney, PCA analysis was performed. Using multivariate PCA analysis, we found that PC1 with the linear combination of parameter coefficients (kidney volume, ADC, D, RBF and Parsimonious F_T_) shown in Table 2 was the best identifier for classification between injured and healthy kidney. The change in kidney volume with AUC of 0.96 raises the validity of using a multi-parametric approach where the AUC was only slightly better at 0.97. However, Hueper et al. reported that changes in kidney volume is influenced by the duration of renal artery clamping^30^. Since determining the exact occlusion time in pathology (e.g., kidney transplant, arterial stenosis) is often impossible, it is crucial to adopt a multiparametric approach instead. It has also been established that kidney volume loss is irreversible and present in several kidney pathophysiological process and is not exclusive to kidney ischemia–reperfusion (IR) injury^59–62^. In the injured kidney, while the volume shows progressive reduction, simultaneously RBF & GFR values show improvement (Fig. 5) on day 15 which is common in AKI. This necessitates the use of multiparametric data for comprehensive monitoring of kidney injury/recovery. Hence, relying solely on kidney volume for diagnosis may lead to errors.

Both ASL and DCE-MRI can provide a measurement of renal blood flow in healthy and diseased kidneys. However, DCE-MRI often overestimates perfusion values^44^ and has lower reproducibility^63^. While some studies report good agreement between perfusion values obtained from ASL and DCE-MRI^64,65^, others have found nontrivial differences and variations between the two methods^44,66^. Additionally, deriving blood flow (RBF) from DCE-MRI necessitates the fitting of PK models, which can introduce complications such as fitting errors and variability in the estimated RBF. We highlight that both ASL and DCE-MRI are important for our work. ASL is a technique which does not require any exogenous CA and has its advantage over the DCE-MRI as it can be used for repeated measurement and can also be used for people with GFR < 30 ml/min. On the other hand, DCE-MRI has the potential to estimate GFR which is the paramount parameter to monitor kidney function. Therefore, we believe that monitoring multiple functional parameters is essential for assessing the progression of AKI pathophysiology and treatment response.

Although the multi-parametric MRI approach aided in differentiating injured from healthy kidney and we could also assess longitudinal changes in MR derived quantitative parameters in assessing progressive kidney damage/normalization in the IRI model, our study has certain limitations. The role of medullary hypoxia as a contributing factor to long term kidney damage is well known and researchers have used BOLD and R_2_^*^ mapping for monitoring medullary hypoxia^67–70^. We have not measured this as addition of BOLD and R2* mapping would have led to additional scan time and the fact that there is no consensus about the best combination of parameters that could have been used for monitoring AKI, we chose to use DCE-MRI, ASL, and DWI-MRI for this study. In our study, we consciously avoided evaluating the medulla since IRI originates as an obstruction to blood flow in the tissue and since all the vasculature is present in the cortex, monitoring the cortex was given higher priority over the medulla. Additionally, due to the loss of the kidney cortex brush border, it was difficult to delineate the medulla from the cortex in the MRI images. Also, our post processing pipeline doesn’t include segmentation of the medulla region. In future work, we plan to implement automated segmentation of the cortex, medulla, and pelvis for better monitoring of the pathophysiology. The constraint of focusing the ROI exclusively on the kidney cortex for ASL and DWI presents a potential obstacle to comprehensive assessment of renal health as it excludes the medulla. Existing literature indicates significant differences in ADC values between injured and healthy medulla, emphasizing the importance of including this region^24,25^. There is also a potential variability of injury and recovery within each individual animal, despite a strict and standardized procedure of renal clamping and anesthesia time^71–73^. There are logistical limitations to the application of multiparametric MRI in the clinical setting of AKI, especially during acute phase. However, the injury and recovery times may be different in a mouse than in a patient. In addition, as shown in our study, the multi-parametric approach can determine changes due to AKI even during the recovery phase and hence we believe that this multi-parametric approach might still be useful in the clinic. In addition, as reasoned above, the multi-parametric approach (sans the DCE-MRI) might also be useful for patients who suffer from nephrotoxicity where the GFR in humans is < 30 ml/min, and hence cannot be given Gd contrast agents.

## Conclusion

This study demonstrates the efficiency of a parsimonious model approach for calculation of GFR from DCE-MRI, offering a promising avenue for non-invasive monitoring of renal health. The differentiation between healthy and injured kidney further establishes the potential of multiparametric MRI in monitoring renal health.

## Materials & methods

### Animals

All animal experiments were performed under a Project License (PPL 70/8741) granted by the Home Office under the Animals (Scientific Procedures) Act 1986 and were approved by the Institutional animal welfare and ethical review body (AWERB). All experiments were performed in accordance with guidelines and regulations by University of Liverpool ethics committee. ARRIVE guidelines were followed to report animal experiments. Albino Black 6 (C57BL/6) mice (Charles River) aged 8–10 weeks were used (n = 19). Mice were housed in individually ventilated cages in a 12 h light/dark cycle. They had ad libitum access to food and water and were acclimatized for 1 week before being entered into the study.

## Transcutaneous GFR measurement

Transcutaneous GFR was measured from ten healthy Albino Black 6 (C57BL/6) mice (Age: 8–10 weeks, weight 25–30 g) for cross-validating the GFR values obtained from DCE-MRI. The measurements were always performed immediately after MR imaging sessions (see below for details). The transcutaneous GFR was measured as described by Scarfe et al.^74^. Briefly, under 1.5% isoflurane anaesthesia, the transcutaneous device (Medibeacon™, Mannheim) was attached to the depilated skin (under isoflurane anesthesia) on the back of the mouse. After recovery from anesthesia, FITC-Sinistrin was injected at 0.15 mg per gram body weight through the tail vein, and the transcutaneous fluorescence reading was recorded by the device for 90 min while the animal was allowed to move freely in its home cage. The device was removed from the animal after 90 min and the data from the device was analyzed by Studio software (V2, Medibeacon™, Mannheim) using a 3-Compartment model with baseline correction and smoothing^18^. A schematic of the experimental design is presented in supplementary Fig. 1.

## Unilateral ischemia reperfusion injury

After validation of the DCE-MRI based GFR values on 10 healthy mice, a separate cohort of nine mice (8–10 weeks old, 25–30 g) were used for the ischemia reperfusion injury (IRI) experiment. Unilateral IRI was induced by clamping the right renal pedicle for 40 min, as described by Harwood et al.^75^. Mice were anesthetised using 1.5% isoflurane for 30 min before the commencement of surgery. Animals were placed on a rectal probe-controlled feedback regulated heat pad with the body temperature set at 37 °C. A dorsal incision was made on the skin to expose the kidney, and an atraumatic vascular clamp was placed on the renal pedicle for 40 min. After removing the clamp, the muscle and skin were closed with sutures and the animal recovered in a warmed chamber for 30 min. Longitudinal imaging studies were performed at baseline, 1 day post-IRI surgery, and 15 days post-IRI surgery. A schematic of the experimental design is presented in supplementary Fig. 1.

## MR imaging

MR imaging was performed on a 9.4 T Bruker scanner using an 86 mm birdcage transmit coil and a four-channel surface receive coil. The mouse tail vein was catheterized using a polytetrafluoroethylene catheter with a 30-gauge needle. The mouse was then placed in a supine position, with the coil covering the lower back of the animal such that it can cover both kidneys. A rectal temperature probe and respiratory pad were used to monitor body temperature and respiration rate. After initial localizer images, a multi-slice T2 weighted (Turbo RARE) sequence was used to measure the kidney volume with the following parameters: matrix size 256 X 128, a field of view of 40 X 20 mm and slice thickness of 1 mm. TE and TR were 10.15 ms and 1112.06 ms, respectively, with eight echoes and eight averages. For T1 mapping and ASL, a Flow-sensitive Alternating Inversion Recovery – Rapid Acquisition with Relaxation Enhancement (FAIR-RARE) sequence was used with 14 inversion times (6.60, 11.69, 20.68, 36.60, 64.75, 114.56, 202.70, 358.63, 634.51, 1122.61, 1986.19, 3514.07, 6217.30, 11000 ms, with matrix size of 128 X 64 and a slice thickness of 1 mm. IVIM-DWI was performed based on a diffusion-weighted spin-echo echoplanar imaging (EPI) sequence with 13 b values (20, 30, 40, 50, 60, 80, 140, 230, 350, 570, 840, 1120, and 1460 s/mm2 ) in three orthogonal directions and a TR = 1100 ms and TE = 35 ms. A Multiple Gradient Echo sequence was used for single-slice DCE imaging with a matrix size of 192 × 96 and FOV of 40 X20 mm, and slice thickness of 1 mm, Flip angle was 25° TE1 and TE2 were 1.26 and 6.99 ms, and TR of 15.43 ms leading to a temporal resolution of 987 ms. Pre-contrast T1 weighted images were acquired for two minutes before injecting a bolus of Gd-BOPTA (50 μl, 0.1 mmol/kg).

## Mathematical modelling

To generate the quantitative parametric maps from the DCE-MRI data, the following PK models were used:

### Patlak model

As described by Sourbron et al.^35^, the Patlak model shown in Eq. (1) was used

1 $$C\left( t \right) = V_{p} C_{p} \left( t \right) + F_{T} *C_{p} \left( t \right)$$

where * is the convolution operator, $C\left(t\right)$ is the total gadolinium-BOPTA concentration, ${V}_{p}$ is the plasma volume, ${F}_{T}$ is the tubular flow and ${C}_{p}\left(t\right)$ is the plasma concentration of gadolinium BOPTA.

### Two compartment filtration model

The two-compartment filtration Model (2CFM) was implemented as described in Annet et al.^42^.This model builds on the Patlak model and is described by Eq. (2):

2 $$C\left( t \right) = V_{p} C_{p} \left( t \right) + F_{T} {\text{e}}^{{\frac{ - t}{{T_{T} }}}} * C_{p} \left( t \right)$$

where * is the convolution operator, ${T}_{T}$ is the mean transit time of the tracer (Gd-BOPTA) in the tubular compartment, while the other parameters are the same as in Eq. (1).

### Two compartment filtration model with outflow

The two Compartment Filtration Model with Outflow (2CFM-O) model was implemented as described in Jiang et al.^45^. Briefly, in this model, ${C}_{0}\left(t\right)$ is the arterial input function calculated from gadolinium concentration in the abdominal aorta plasma. There are three compartments in this model: ${C}_{1}\left(t\right)$ which is the gadolinium-BOPTA concentration in renal plasma space, ${C}_{2}\left(t\right)$ is the gadolinium concentration in the tubules distributed over the whole kidney, ${C}_{3}\left(t\right)$ is the gadolinium concentration in the inner medullary papilla (IMP) (calculated from drawing a region of interest (ROI) over the IMP and averaging the signal), ${k}_{1}$ is the perfusion rate constant, ${k}_{2}$ is the gadolinium efflux rate constant.

3 $$\frac{d{C}_{1}\left(t\right)}{dt}={k}_{1}\left({C}_{0}\left(t\right)-{C}_{1}\left(t\right)\right)$$

$$\frac{d{C}_{2}\left(t\right)}{dt}={F}_{T}{C}_{1}\left(t\right)-{k}_{2}{C}_{3}\left(t\right)$$

### Parsimonious model for fitting the DCE-MRI data

A parsimonious model using the Akaike Information Criterion (AIC) from the three different PK models used to analyze the DCE-MRI data was used to obtain the best estimate of the kidney filtration rate (F_T_) for each individual imaging voxel for measuring GFR and thereby monitoring kidney health.

The akaike information criterion (AIC) serves as a statistical measure to evaluate the appropriateness of a model by considering both its goodness of fit and complexity. It establishes a balance between accurately representing the data and avoiding unnecessary model complexity to prevent overfitting. A lower AIC value indicates a more favorable compromise between fitting the model to the data and maintaining simplicity. In model comparison, the model with the lowest AIC is preferred for providing a good fit with a minimal number of parameters. This criterion proves particularly beneficial in scenarios with multiple model options, helping to choose the one that optimally balances accuracy and simplicity. The application of Akaike criterion-based model selection is prevalent in studies involving DCE-MRI for optimizing PK models^76–79^.

## Data analysis

MRI data analysis was performed using in-house developed MATLAB routines. An ROI was drawn manually around the renal mass to calculate the renal volume from T2 weighted images. For DWI and ASL analysis, ROIs were drawn around the renal cortex. We focused on the kidney cortex for assessing kidney function given that both the vasculature and the filtration unit (nephrons) reside in the cortical region. DWI data was analysed using both the monoexponential model to generate apparent diffusion coefficient (ADC) maps as well as the biexponential intra voxel incoherent motion (IVIM) model. The IVIM model was used to calculate D or true diffusion, D* or the pseudodiffusion coefficient, and f or the perfusion fraction. For DCE-MRI analysis, ROIs were placed around the renal parenchyma for Patlak and 2CFM model^56^, adding an ROI on the Inner Medullary Papillae (IMP) for 2CFM-O model^45^. Initial fitting parameter values were selected based on literature values^56^.

Statistical analysis was performed using Origin Pro. Univariate analysis was performed using two-way ANOVA with Bonferroni correction at p < 0.05. For ANOVA with Bonferroni correction, we compared the left kidney to the right kidney on baseline (when both kidneys are healthy), Day 1 after IRI injury (right /injured vs left/contralateral), and on Day 15 after IRI. Univariate Receiver operating characteristics was used to identify best parameters to distinguish between injured and healthy kidney. Multivariate analysis was done using Principal Component Analysis (PCA) to identify the contribution of each parameter identified by ROC analysis to create a classifier to efficiently distinguish between injured and healthy kidney. Baseline data from the left and right kidney was used as healthy control while data from the right IRI kidneys at days 1 and 15 of imaging were used to assess the injury response.

### Supplementary Information

Supplementary Figure 1.

## Data Availability

All data are incorporated into the article and its online supplementary information. Raw data values are available upon request.
