# Supplementary material for: Multiparametric MRI based assessment of kidney injury in a mouse model of ischemia reperfusion injury
Source: Sci Rep. 2024 Aug 27;14:19922. doi: 10.1038/s41598-024-70401-x (PMC11358484; doi:10.1038/s41598-024-70401-x)
Supplement: Supplementary file 1 — Supplementary Figure 1. [file 41598_2024_70401_MOESM1_ESM.pdf]

Multiparametric MRI based assessment of kidney injury in a mouse model of ischemia reperfusion injury

Soham Mukherjee, Sourav Bhaduri , Rachel Harwood, Patricia Murray, Bettina Wilm, Rachel Bearon, Harish Poptani

Supplementary:

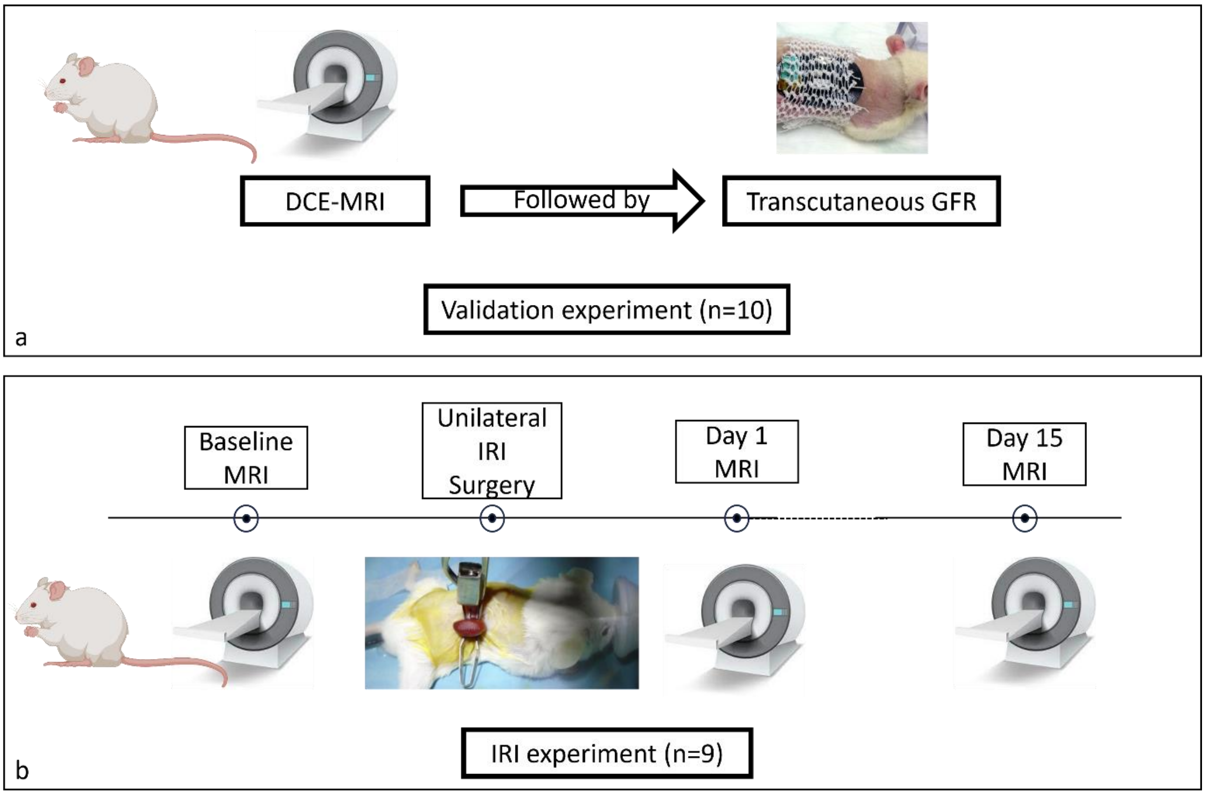

Supplementary Figure 1: Shows the experiment plan. a) validation experiment with 10 healthy mice undergoing DCE-MRI for GFR calculation using parsimonious model followed by GFR measurement using transcutaneous device. b) IRI experiment with 9 mice undergoing baseline MRI scan followed by unilateral IRI surgery on the right kidney on day 0. The mice were then scanned on day 1 and day 15 after IRI surgery.
